# Supplementary material for: Structural Design, Synthesis and Antioxidant, Antileishmania, Anti-Inflammatory and Anticancer Activities of a Novel Quercetin Acetylated Derivative
Source: Molecules. 2021 Nov 17;26(22):6923. doi: 10.3390/molecules26226923 (PMC8623808; doi:10.3390/molecules26226923)
Supplement: Supplementary file 1 [file molecules-26-06923-s001.zip › molecules-1449056-supplementary.pdf]

molecules

MDPI

Article

Structural Design, Synthesis and Antioxidant, Antileishmania, Anti-Inflammatory and Anticancer Activities of a Novel Quercetin Acetylated Derivative

Saul Vislei Simões da Silva[1], Orlando Maia Barboza[1], Jéssica Teles Souza[2], Érica Novaes Soares[2], Cleonice Creusa dos Santos[2], Luciano Vasconcellos Pacheco[1,3], Ivanilson Pimenta Santos[3], Milena Botelho Pereira Soares[3,4], Cássio Santana Meira[1,3,4], Silvia Lima Costa[2], Victor Diógenes Amaral da Silva[2], Lourenço Luís Botelho de Santana[1], and Aníbal de Freitas Santos Júnior[1,*]

[1] Life Sciences Department, University of Bahia State, 41150-000, Salvador, BA, Brazil; saulvislei@gmail.com (S.V.S.S.); orlandmb0@gmail.com (O.M.B.); lucianofcd@hotmail.com (L.V.P.); cassio.meira@fieb.org.br (C.S.M.); lourencoluisbotelho@gmail.com (L.L.B.S.); anibaljrr@uol.com.br (A.F.S.J.)

[2] Biochemistry and Biophysics Department, Federal University of Bahia, Laboratory of Neurochemistry and Cell Biology, 40231-300, Salvador, BA, Brazil; telles.jessica@hotmail.com (J.T.S.); ericanovaessoares@gmail.com (E.N.S.); cleonicemev@gmail.com (C.C.S.); costasl@ufba.br (S.L.C.); vdsilva@ufba.br (V.D.A.S.)

[3] Gonçalo Moniz Institute, FIOCRUZ, 40296-710, Salvador, BA, Brazil; lucianofcd@hotmail.com (L.V.P.); eagle_nito@hotmail.com (I.P.S.); milena@bahia.fiocruz.br (M.B.P.S); cassio.meira@fieb.org.br (C.S.M.)

[4] SENAI Institute of Innovation in Health Advanced Systems (CIMATEC ISI SAS), University Center SENAI/CIMATEC, Salvador, BA, Brazil, 41650-010, Salvador, BA, Brazil; milena@bahia.fiocruz.br (M.B.P.S); cassio.meira@fieb.org.br (C.S.M.)

* Correspondence: anibaljrr@uol.com.br; afjunior@uneb.br (A.F.S.J.) Tel.: +55 71 3117-5313

Citation: da Silva, S.V.S.; Barboza, O.M.; Souza, J.T.; Soares, É.N.; dos Santos, C.C.; Pacheco, L.V.; Santos, I.P.; Magalhães, T.B.d.S.; Soares, M.B.P.; Guimarães, E.T.; et al. Structural Design, Synthesis, and Antioxidant, Antileishmania, Anti-Inflammatory and Anticancer Activities of a Novel Quercetin Acetylated Derivative. *Molecules* **2021**, *26*, 6923. https://doi.org/10.3390/molecules26226923

Academic Editors: Jianbo Xiao and H. P. Vasantha Rupasinghe

Received: 19 October 2021
Accepted: 14 November 2021
Published: 17 November 2021

Publisher's Note: MDPI stays neutral with regard to jurisdictional claims in published maps and institutional affiliations.

Copyright: © 2021 by the authors. Licensee MDPI, Basel, Switzerland. This article is an open access article distributed under the terms and conditions of the Creative Commons Attribution (CC BY) license (https://creativecommons.org/licenses/by/4.0/).

Supplementary information – Figures / Analysis by ^{1}H and ^{13}C NMR

^{1}H NMR analyzes confirmed the structural skeleton of the quercetin molecule with five singlet signals in the highest range of the spectrum, corresponding to the five hydrogens linked to the hydroxyls (Figure S1). Noteworthy is the characteristic signal of the most electronegative hydroxyl hydrogen, linked to carbon 5, corresponding to the region of approximately 12.48 ppm of the spectrum (Figure S2), and the intermediate regions of the spectrum between 6 and 8 ppm, corresponding to singlets and doublets of aromatic hydrogens, characteristic of quercetin ^{1}H NMR (500MHz, DMSO D6): 12.48 (1H, s, 5-OH); 9.56 (1H, s, 5-OH); 9.32 (1H, s, 4´-OH); 9.28 (1H, s, 3-OH); 7.68 (1H, brd, J= 2.5 Hz); 7.54 (1H, dd, J= 10.5 Hz, J= 8.5 Hz or 6.90 (2H, brd, J=8.5 Hz); 6.41 (1H, brd, J= 2 Hz); 6.19 (1H, brd, J= 2.5 Hz).

^{13}C NMR spectrum showed the carbonyl carbon, characteristic of flavonols, with a signal in the spectrum at 175.81 ppm, and the remainder of the carbons with signals corresponding to the range of aromatic carbons in the spectrum, characteristic of quercetin, in addition to the absence of carbons in the smaller region of the spectrum, from aliphatic carbons (Figure S3): ^{13}C NMR (126 MHz, DMSO D6) δ 175.81; 163.85; 160.69; 156.11; 147.67; 146.78; 145.03; 135.69; 121.93; 119.94; 115.57; 115.04; 102.99; 98.15 and 93.32.

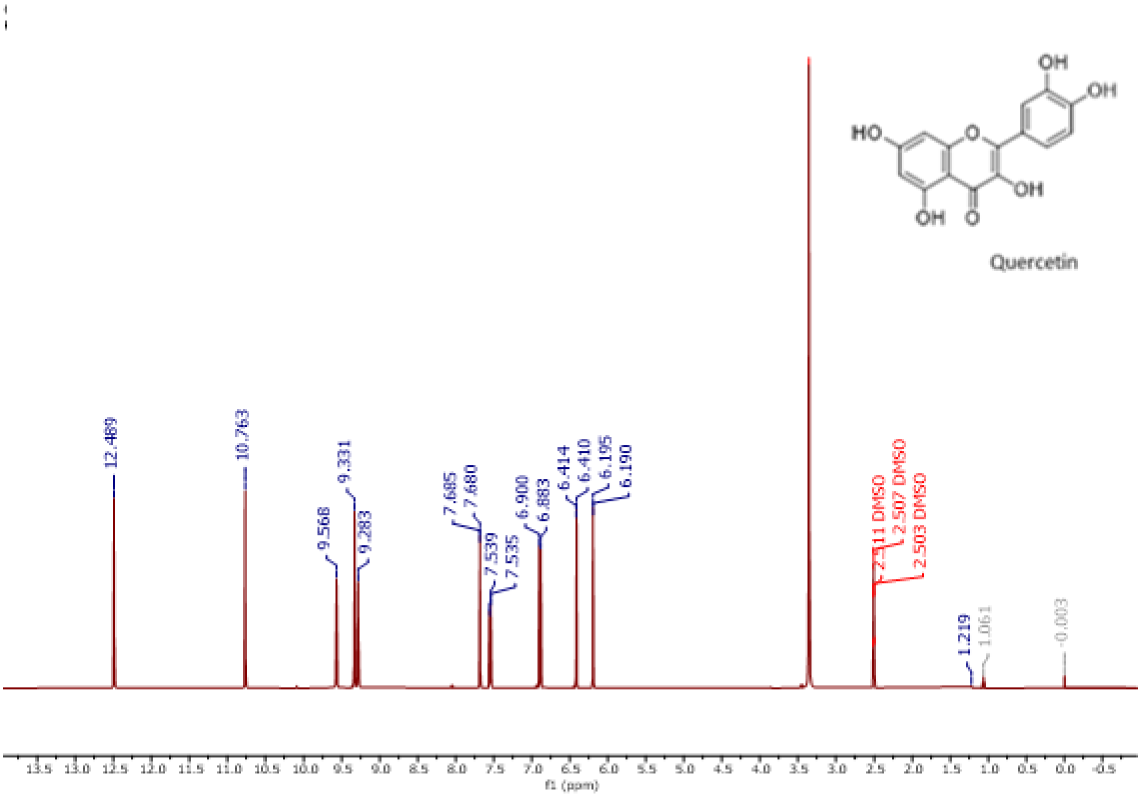

Figure S1. Complete ¹H NMR spectrum of Quercetin (Q)

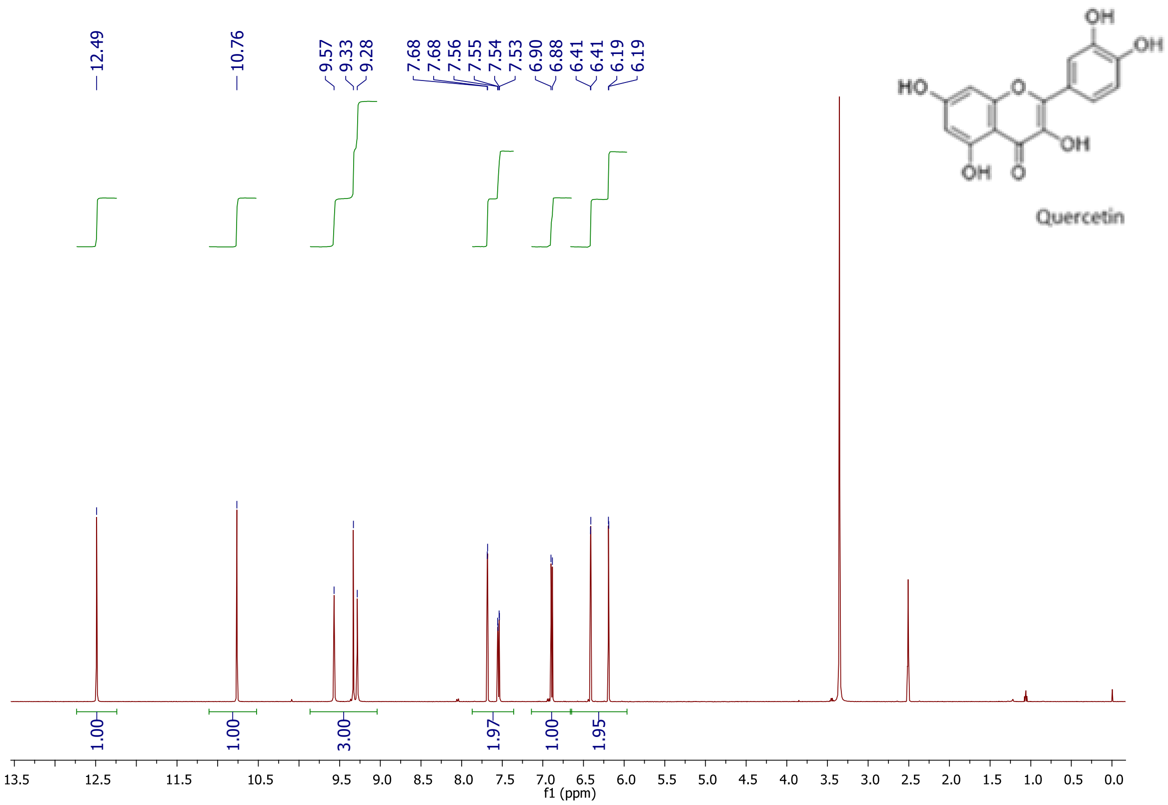

Figure S2. Expansion of Quercetin (Q) ¹H NMR Spectrum

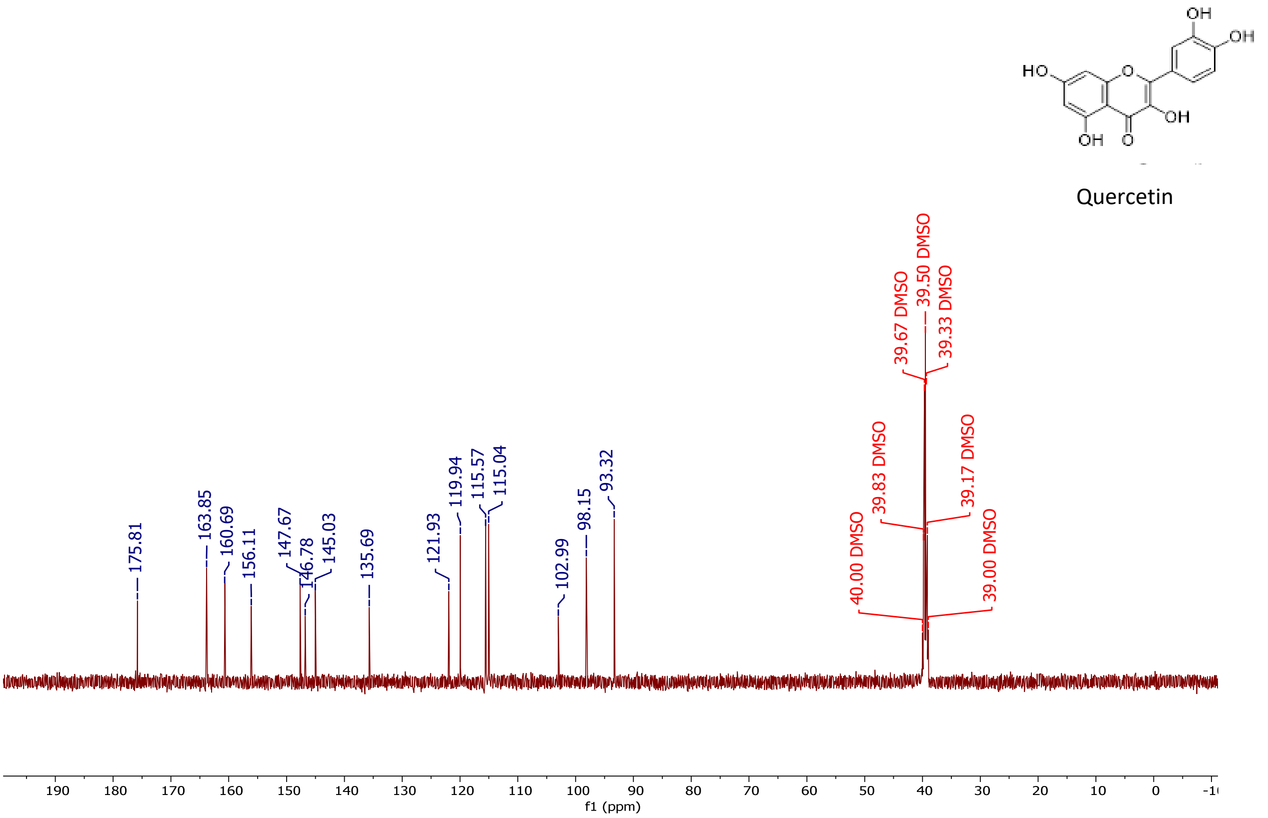

Figure S3. Complete ^{13}C NMR spectrum of Quercetin (Q)

For the compound obtained (Q5), the analysis of the ^{1}H NMR spectra showed a pattern that showed that it was the quercetin pentaacetate analogue (Figure S4). Signals of the product obtained showed total acetylation, with the disappearance of the characteristic singlets of hydroxyls above 9 ppm in the ^{1}H NMR spectrum, and the appearance of large and characteristic signals of methyls in the aliphatic regions of the spectrum, between 2 and 3 ppm (Figures S5 and S6): ^{1}H NMR (500 MHz, $CDCl_3$) 2.34 (6 H, s, $-OCOCH_3$); 2.35 (3 H, s, $-OCOCH_3$); 2.35 (3 H, s, $-OCOCH_3$); 2.44 (3 H, s, $-OCOCH_3$); 6.88 (1 H, d, J = 2.5 Hz); 7.34 (1 H, d, J =2.0 Hz); 7.36 (1 H, d, J =8.5); 7.70 (1 H d, J = 2.0 Hz); 7.73 (1H, dd, J1=8.5 Hz, J2 =2.0 Hz).

For the ^{13}C NMR spectrum, the product obtained showed an increase in the representative signals of the ester carbonyls close to 170 ppm and the appearance of signals between 20 and 21 ppm corresponding to the chemical shifts of the five aliphatic carbons of the methyls, verified through the integral of the signals (Figures S7, S8 and S9): ^{13}C NMR (126 MHz, $CDCl_3$) δ 170.04; 169.24; 167.86; 167.79; 156.87; 154.28; 150.43; 144.40; 142.22; 127.78; 126.42; 124.01; 123.93; 123.85; 113.89; 108.96; 21.16; 21.02; 20.65 and 20.49.

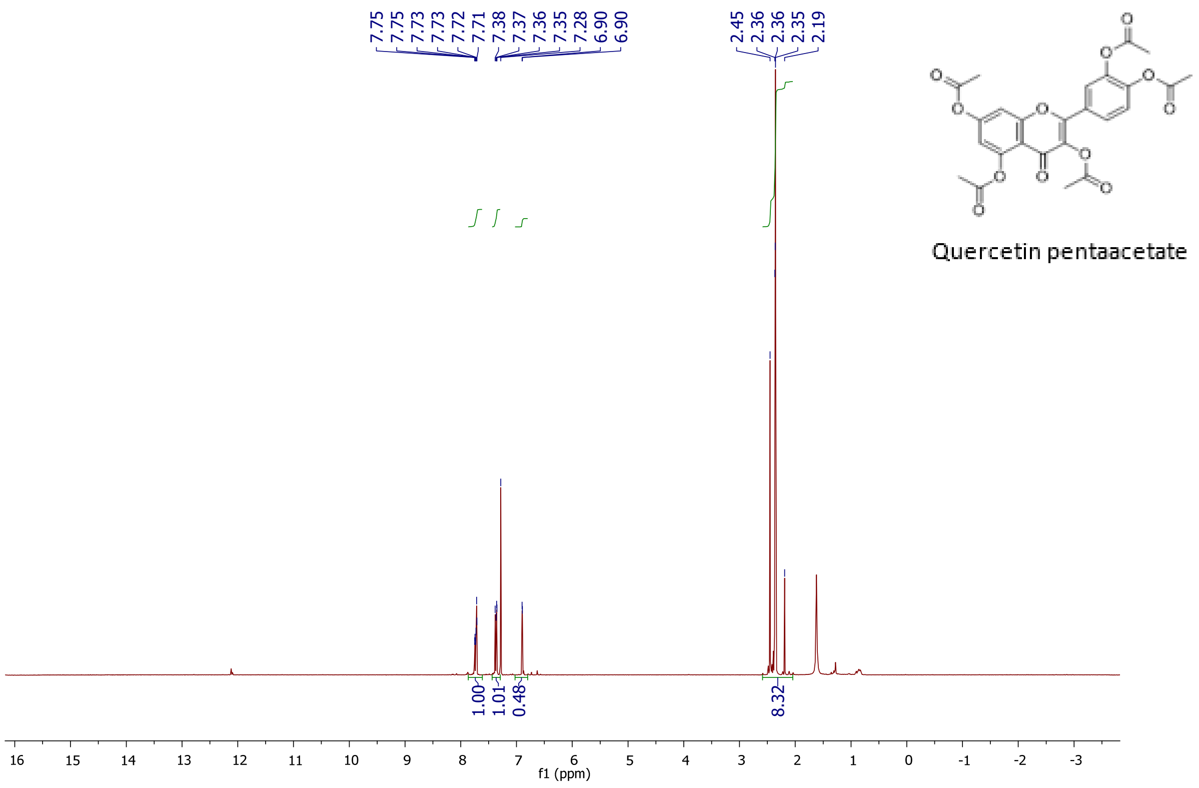

Figure S4. Complete ^{1}H NMR spectrum of the obtained compound - Quercetin pentaacetate analogue (Q5)

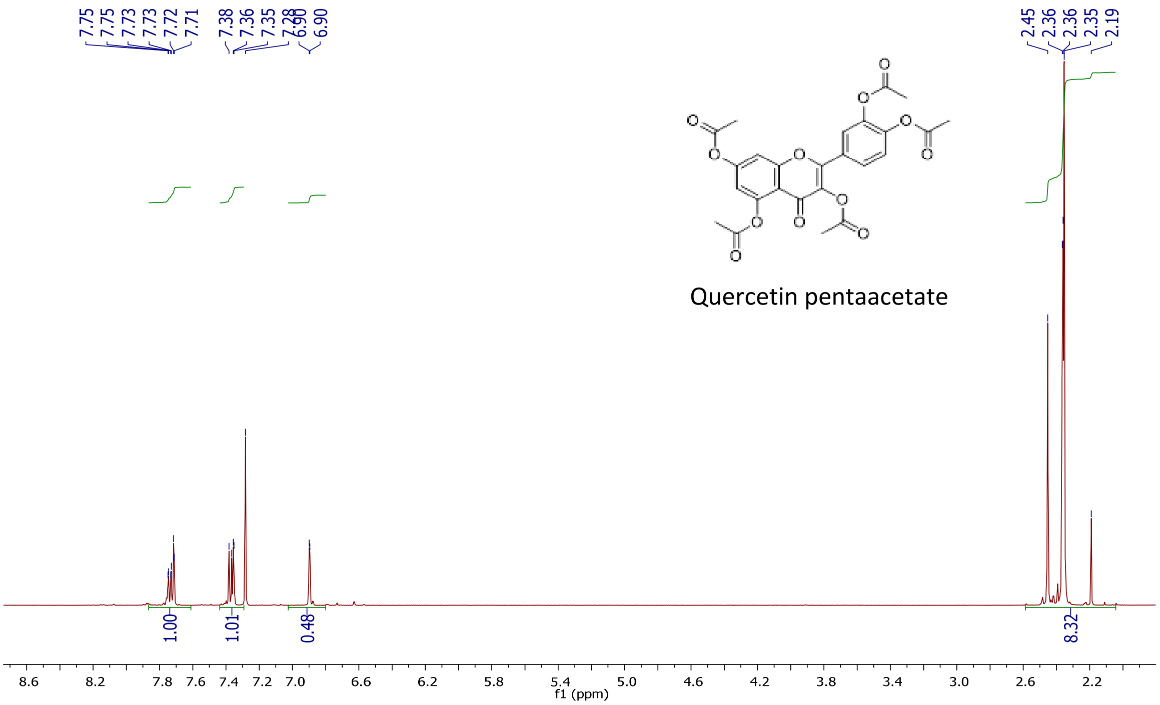

Figure S5. Expansion of the ^{1}H NMR spectrum of the obtained compound – Quercetin pentaacetate analogue (Q5)

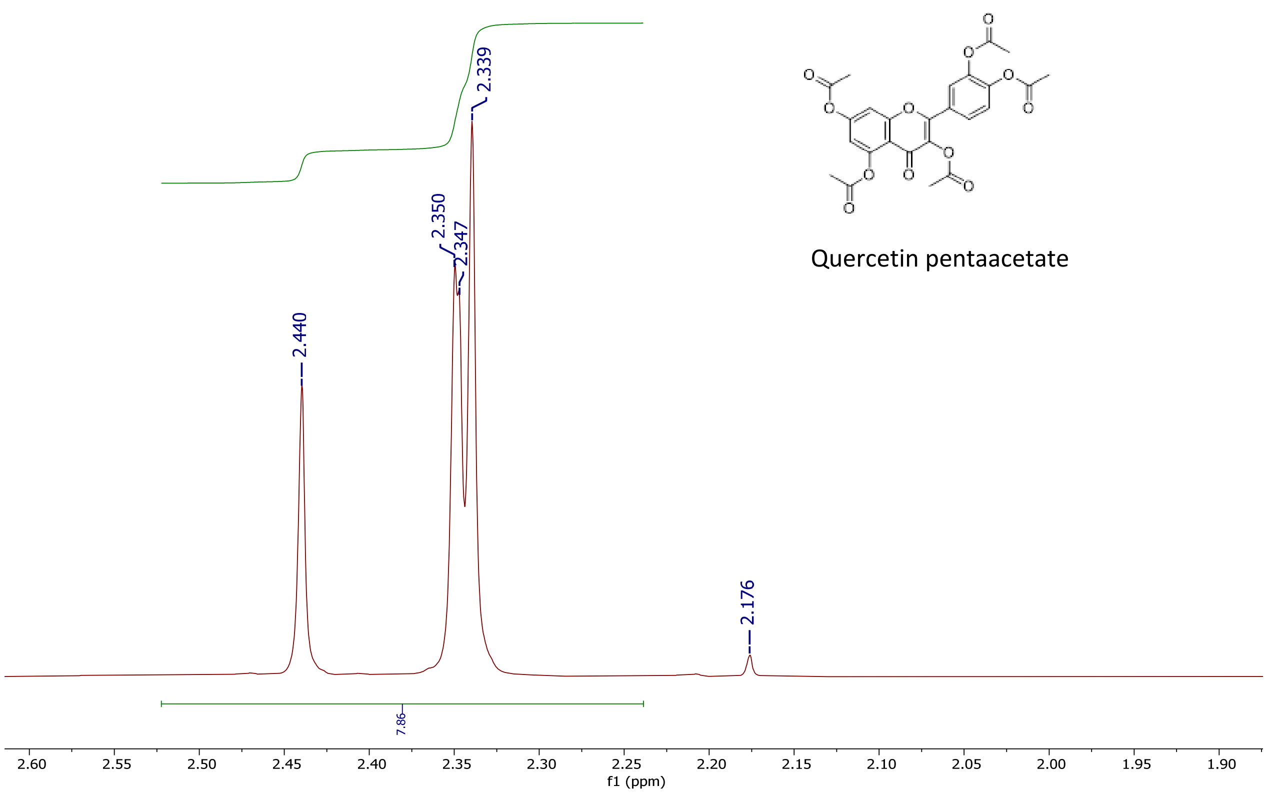

Figure S6. Expansion of the more aliphatic region of the ^{1}H NMR spectrum of the obtained compound – Quercetin pentaacetate analogue (Q5)

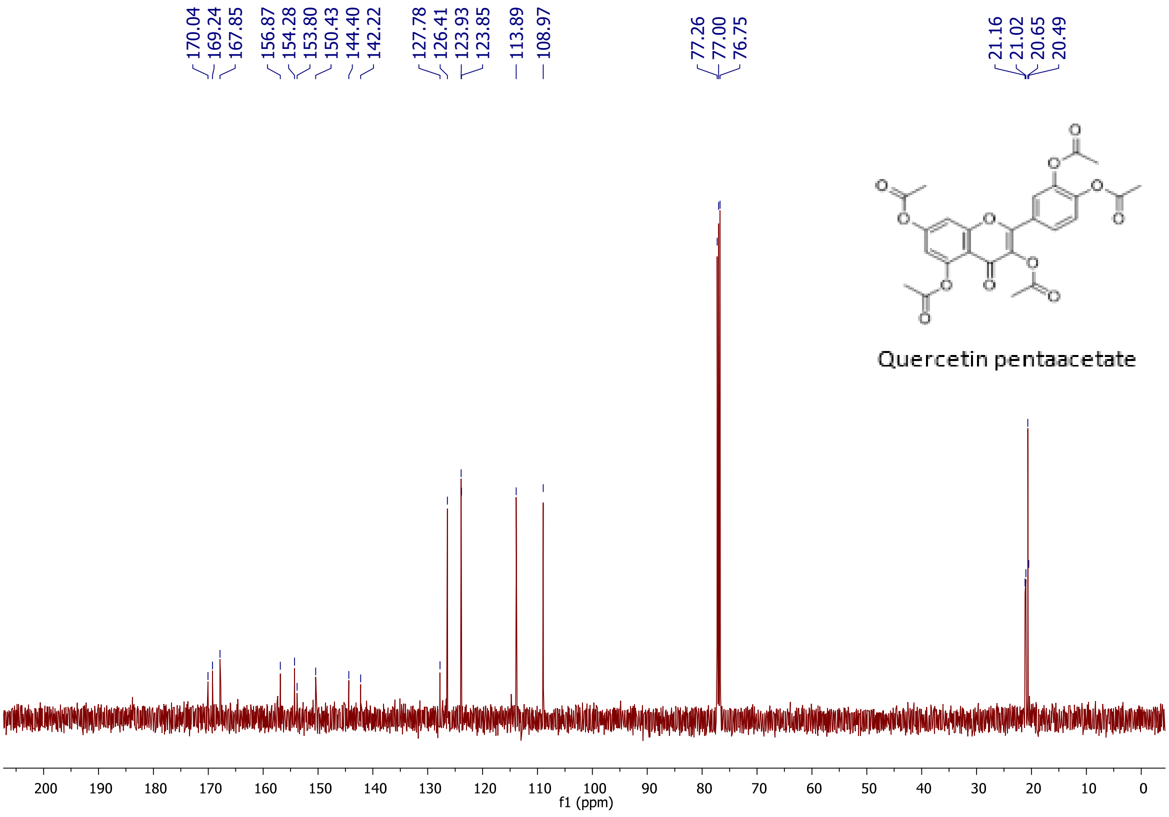

Figure S7. Complete ^{13}C NMR spectrum of the obtained compound – Quercetin pentaacetate analogue (Q5)

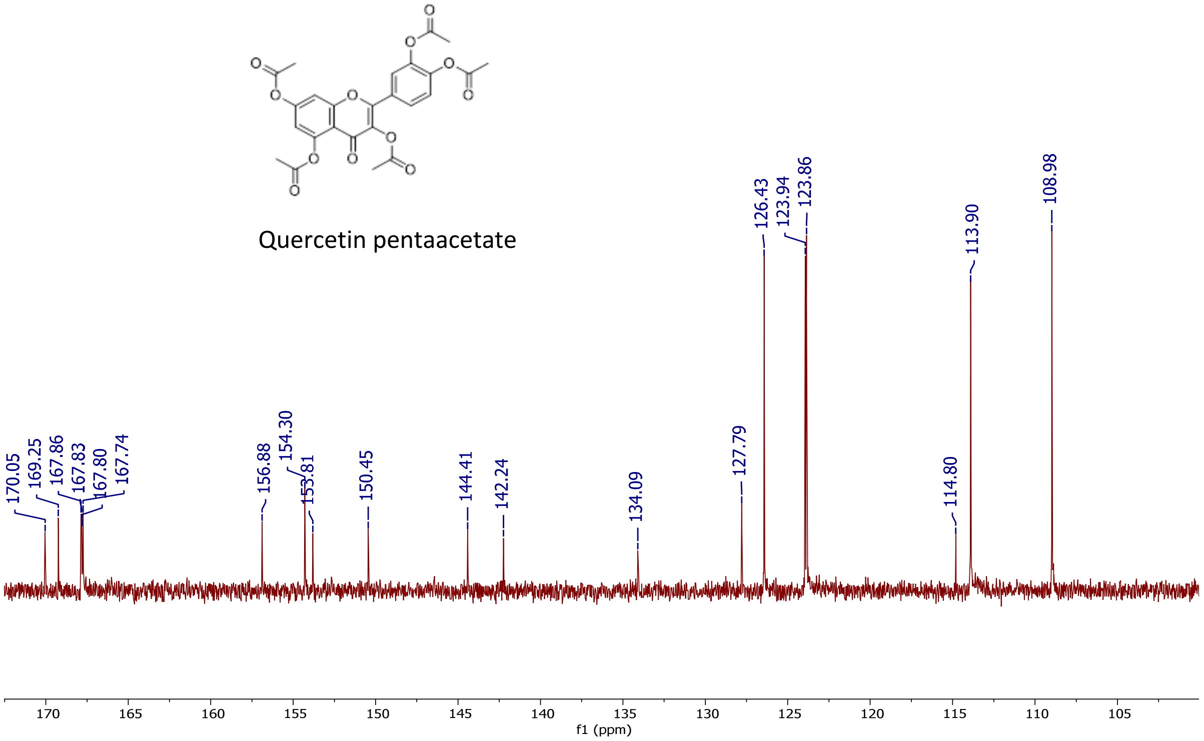

Figure S8. Expansion of the ^{13}C NMR spectrum of the obtained compound – Quercetin pentaacetate analogue (Q5)

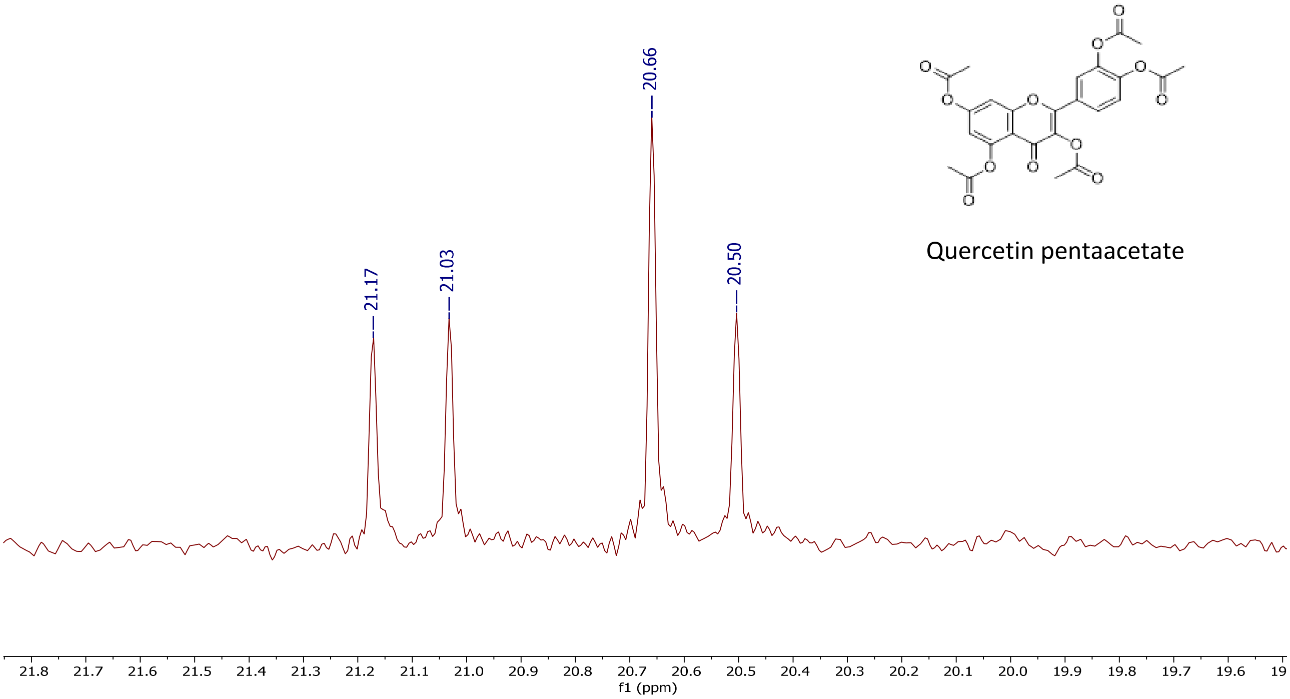

Figure S9. Expansion of the aliphatic region of the ^{13}C NMR spectrum of the obtained compound – Quercetin pentaacetate analogue (Q5)
